# Effects of Curcumin on the Renal Toxicity Induced by Ochratoxin A in Rats

**DOI:** 10.3390/antiox9040332

**Published:** 2020-04-18

**Authors:** Sara Damiano, Emanuela Andretta, Consiglia Longobardi, Francesco Prisco, Orlando Paciello, Caterina Squillacioti, Nicola Mirabella, Salvatore Florio, Roberto Ciarcia

**Affiliations:** 1 Department of Veterinary Medicine and Animal Productions, University of Naples “Federico II”, Naples, Via Delpino, 1, 80137 Napoli, Italy; emanuelaandretta94@gmail.com (E.A.); francesco.prisco@unina.it (F.P.); orlando.paciello@unina.it (O.P.); caterina.squillacioti@unina.it (C.S.); nicola.mirabella@unina.it (N.M.); salvatore.florio@unina.it (S.F.); roberto.ciarcia@unina.it (R.C.); 2 Department of Mental, Physical Health and Preventive Medicine, University of Campania “Luigi Vanvitelli” Naples, Largo Madonna delle Grazie, 1, 80138 Napoli, Italy; consiglia.longobardi@unicampania.it

**Keywords:** ochratoxin-A, curcumin, oxidative stress, kidney, toxicity

## Abstract

Ochratoxin A (OTA) is a powerful nephrotoxin and the severity of its damage to kidneys depends on both the dose and duration of exposure. According to the scientific data currently available, the mechanism of action still is not completely clarified, but it is supposed that oxidative stress is responsible for OTA-induced nephrotoxicity. Bioactive compound use has emerged as a potential approach to reduce chronic renal failure. Therefore, curcumin (CURC), due to its therapeutic effects, has been chosen for our study to reduce the toxic renal effects induced by OTA. CURC effects are examined in Sprague Dawley rats treated with CURC (100 mg/kg), alone or in combination with OTA (0.5 mg/kg), by gavage daily for 14 days. The end result of the experiment finds rats treated with OTA show alterations in biochemical and oxidative stress parameters in the kidney, related to a decrease in the Glomerular Filtration Rate (GFR). Conversely, the administration of CURC attenuates oxidative stress and prevents glomerular hyperfiltration versus the OTA group. Furthermore, kidney histological tests show a reduction in glomerular and tubular damage, inflammation and tubulointerstitial fibrosis. This study shows that CURC can mitigate OTA–induced oxidative damage in the kidneys of rats.

## 1. Introduction

Ochratoxin A (OTA) is a mycotoxin derived from the secondary metabolism of different fungi genera, in particular it originates from *Aspergillus ochraceus, A. carbonarius*, *A. niger and Penicillium verrucosum*, in tropical areas and in temperate climate regions, respectively [1]. OTA is generally found in all types of cereals and derived products, but also in coffee, cocoa, grapes, soy, spices, legumes, nuts, licorice, wine, and beer [2,3,4]. OTA is often the cause of chronic toxicity, due to the prolonged intake of its small amounts, and manifests itself with carcinogenic, genotoxic, teratogenic, nephrotoxic and immunosuppressive effects [5,6,7] both on humans and numerous animal species. Previous studies have found that its presence in food for human use can induce chronic kidney diseases, such as Endemic Balkan Nephropathy which leads, in a period of 6–10 years, to irreversible renal failure [8,9]. Added to an indirect contamination, which occurs with the ingestion by animals of naturally contaminated feed, subsequent passage, and accumulation of OTA in tissues (carry over), a direct contamination is possible. This kind of contamination is linked to the growth of molds producing OTA on the meat products during the seasoning or the addition of contaminated spices during the product processing [10]. Among the production animals, the risk is limited to monogastric species since ruminants can hydrolyze the OTA amide bond by forming a non-toxic compound [11]. Particularly, pigs are the most sensitive to the accumulation of OTA, which is deposited in the tissue level following the kidney > liver > muscle > fat model [12]. Therefore, the European Communities Commission recommends a guide value of OTA that should not exceed 0.05 mg/kg in complementary and complete feeds intended for pigs [11]. Indeed, intoxication by OTA is considered not only a sanitary, but also an economic problem. The chronic OTA-intake through contaminated feeds causes significant production losses in the zootechnical environment linked to the decrease of food intake, production yields, and fertility in animals [13]. The toxic action of OTA mostly affects kidneys, but other organs, such as the liver, and the immune system are involved [14]. Although, even if the effects of OTA are known, the molecular mechanisms that regulate its effects still are not completely clarified. However, it is estimated that oxidative stress and the phlogistic state are responsible for OTA-induced nephrotoxicity. Phytochemicals use, thanks to their anti-inflammatory and antioxidant effects, has emerged as a potential approach to reduce chronic renal failure. Therefore, in this study we evaluate the efficacy of curcumin (CURC) on OTA-induced nephrotoxicity. CURC is a polyphenolic compound that derives from the rhizome of Curcuma longa Linn, belonging to Zingiberaceae family. Several studies demonstrate that CURC has numerous pharmacological antioxidants [15], anti-inflammatory [16], antiviral and antibacterial properties [17], as well as its therapeutic potential effect on tumors [18]. CURC has shown to inhibit inflammatory processes and to be a powerful scavenger of both reactive oxygen species (ROS) and reactive nitrogen species (RNS), as well as to preserve the renal mitochondrial redox balance during acute and chronic nephrotoxicity [19]. CURC has a strong antioxidant action by acting directly on reactive oxygen species, by reducing the levels of anion superoxide (O_2_^−^), peroxynitrite (ONOO^−^), nitric oxide (NO), peroxyl radicals (HO_2_) and hydroxyl radicals (OH^−^) [16]. Particularly, the antioxidant activity of CURC is linked to its phenolic groups which, reacting with ROS, protects cells from oxidative damage. Furthermore, CURC is able, indirectly, to induce the over-expression of antioxidant proteins such as superoxide dismutase (SOD), catalase (CAT) and glutathione peroxidase (GPx). Furthermore, CURC seems to act by increasing the synthesis of glutathione (GSH) levels through the induction of γ-glutamylcysteine ligase (γ-GCL) [19]. The aim of this study is to investigate the effects of CURC against OTA-induced nephrotoxicity by biochemical indices assessment and histological changes in the kidney tissue of rats.

## 2. Materials and Methods

### 2.1. Chemicals

OTA and CURC were purchased from Sigma–Aldrich (Milan, Italy). SOD (Item No. 19160), malondialdehyde (MDA) (Item No. MAK085), GPx (Item No. 38185) and CAT (Item No.CAT100) assay kits were purchased from Sigma–Aldrich (Milan, Italy). The diagnostic kits of creatinine (CREA) and urea nitrogen (BUN) were obtained from Sigma–Aldrich (Milan, Italy). The establishment that supplies the animals was Charles River Laboratories (Milan, Italy).

### 2.2. Ethics Statement

The use and care of the animals in this work was approved by the Institutional Animal Care and Ethics Committee (Approval Number: 487/2018-PR) and carried out in accordance with the associated guidelines EU Directive 2010/63/EU.

### 2.3. Experimental Design and Sample Collection

During this study, 10-week-old (250–270 g), male Sprague Dawley rats, were randomly allocated into four experimental groups (6 rats for each group) experiencing 22 °C temperatures and 12 h day/night cycles. The animals received a standard diet ad libitum. Animals were treated daily for 14 days by gavage as follows: Control group: 2 mL/kg body weight (b.w.) of olive oil; OTA group: 2 mL/kg b.w. of olive oil containing 0.5 mg/kg b.w. of ochratoxin A [7]; CURC group: 2 mL/kg b.w. of olive oil containing 100 mg/kg b.w. of curcumin [20]; OTA (1 mL/kg b.w. of olive oil containing 0.5 mg/kg b.w. of ochratoxin A.) + CURC (1 mL/kg b.w. of olive oil containing 100 mg/kg b.w. of curcumin). The use of olive oil served to improve the stability of curcumin. The duration of the experiment (14 days) was based on our previous work [7,21,22]. Body weights were monitored at three time points: before the treatment (time 0), after 7 and 14 days of treatment. Once the experimental period ended, rats were anesthetized with 2% isoflurane (Isotec 4, Palermo Italy) and, after completely sedated, blood samples were collected from the aortas and immediately centrifuged at 3000 rpm for 15 min at 4 °C. Blood urea nitrogen (BUN) and creatinine (CREA) levels were measured after 14 days of treatment by an auto-chemistry analyzer (PKL PPC 125, Paramedical srl, Salerno, Italy) using commercial diagnostic kits following the manufacturer’s instructions. Following the end of the treatment, animals anesthetized with 2% isoflurane were prepared for the assessment of the clearance of inulin to measure the glomerular filtration rate (GFR) index expressed as ml/min 100 g body weight (b.w.), as previously described in our study [7]. Subsequent to the end of clearance experiments, rats underwent euthanasia by an overdose of 4% isoflurane (Isotec 4, Palermo Italy) and samples of kidney were taken on ice and stored at −80 °C until analyzed for the activity of enzymes involved in oxidative stress and lipid peroxidation, and partially prepared for routine histopathology.

### 2.4. Antioxidants and Lipid Peroxidation Assay

Kidney samples from all groups were collected at day 14 of the treatment. One gram of each kidney sample was added to 9 mL of normal saline 0.9% and homogenized using an electrical tissue homogenizer and centrifuged at 3000 rpm for 15 min at 4 °C. The supernatant was collected and used to evaluate, by a spectrophotometer (Glomax Multi detection system, Promega, Milano, Italy) the SOD, CAT and GPx activity according to previous studies [23,24,25]. These activities were expressed as unit for milligrams of proteins (U/mg of proteins). Malondialdehyde (MDA), a marker of lipid peroxidation, was calculated according to Ensiyeh Ghanizadeh–Kazeroun et al. [26] and following the manufacturer’s instructions. The optical density (OD) of the supernatants was read by a spectrophotometer at a wavelength of 532 nm and was expressed in nmoles of MDA for milligrams of proteins.

### 2.5. Histopathological Studies

Kidneys collected during necropsy of 24 male Sprague Dawley rats (6 rats per group) were fixed in Bouin’s solution for 24 h, subsequently dehydrated in ascending ethyl alcohol, and then embedded in paraffin. Two serial sections at 4 μm were stained with hematoxylin, eosin and with Masson’s trichrome stain then examined and photographed with a light microscope (Nikon eclipse E600, Melville, NY, USA) associated to a microphotography system (Nikon digital camera DMX1200, Melville, NY, USA). The hematoxylin and eosin stained sections were used to score the severity of glomerular damage, tubular damage, inflammation, and the presence of proteinaceous material in Bowman’s spaces and tubule lumen from 0 to 3 (0 = absent, 1 = mild, 2 = moderate, 3 = intense). The severity of the fibrosis was evaluated on the Masson’s trichrome stained section with ImageJ software (National Institutes of Health) as previously described [27].

### 2.6. Statistical Analysis

All the results are expressed as mean ± standard deviation (SD). Analysis of variance ANOVA tests, followed by a Tukey’s test, were used to analyze the differences (GraphPad Software 3.00, San Diego, CA). Each animal group consisted of 6 rats and the experiment was done in triplicate. Values of *p < 0.05 were considered significant.

## 3. Results

### 3.1. Effect of CURC on Body Weight of Rats

The body weight changes in treated rats at 0, 7 and 14 days are presented in Table 1. The oral administration of OTA caused a significant reduction in body weights compared to the Control group at 7 and 14 days of treatment. (−16.1% and −22.2%, respectively). However, the combination treatment with curcumin significantly attenuated the adverse effects of OTA after 14 days of treatment (5.1%).

### 3.2. Biochemical Analyses

Observed in the OTA group was a significant increase in BUN and CREA levels compared to the Control group of +36.7% and +48.1%, respectively, after 14 days of treatment (Table 2). The CURC plus OTA caused a good restoration in BUN and CREA parameters, compared to the OTA group, of 19.5% and 14.2% respectively. (*p < 0.05 versus Control group and #*p* < 0.05 versus OTA group).

### 3.3. Clearance of Inulin

**Figure 1:**
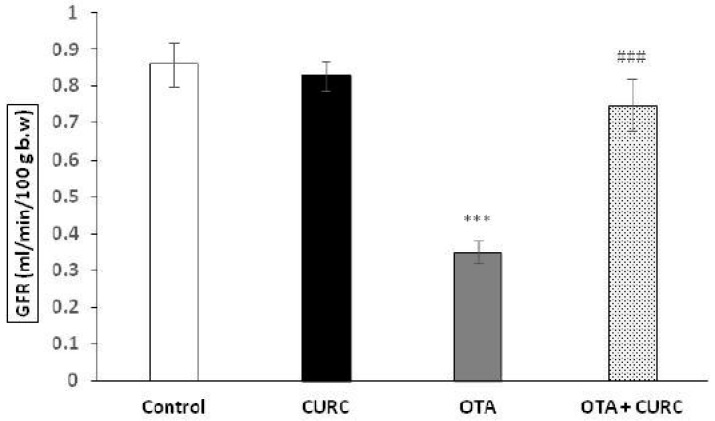
Effects of Curcumin (CURC) on the glomerular filtration rate (GFR), expressed as ml/min 100 g body weight (b.w.) after 14 days of treatment. Control group (Control); Curcumin group (CURC); Ochratoxin A group (OTA); Curcumin plus Ochratoxin A group (CURC + OTA). Data are expressed as mean ± standard deviation (SD) of *n* = 6 rats. OTA treatment significantly decreased GFR, while co-administration with CURC significantly restored this effect (***p < 0.001 versus Control; ###*p* < 0.001 versus OTA).

Figure 1 shows the effect of CURC and OTA treatment on GFR after 14 days of treatment. The OTA group significantly decreased their GFR versus the Control group (0.86 ± 0.06 Control group to 0.35 ± 0.03 OTA group) (***p < 0.001). Instead, the CURC in association with OTA completely prevented this renal hemodynamic alteration. Actually, the GFR value was 0.75 ± 0.07 mL/min in the OTA plus CURC group versus 0.35 ± 0.03 in the OTA group (^#^^##^*p* < 0.001). CURC alone did not induce any change on the GFR in respect to the Control group (0.83 ± 0.04 CURC group versus 0.86 ± 0.06 Control group).

### 3.4. Antioxidant Enzymes SOD, CAT, GPx Activity

**Figure 2:**
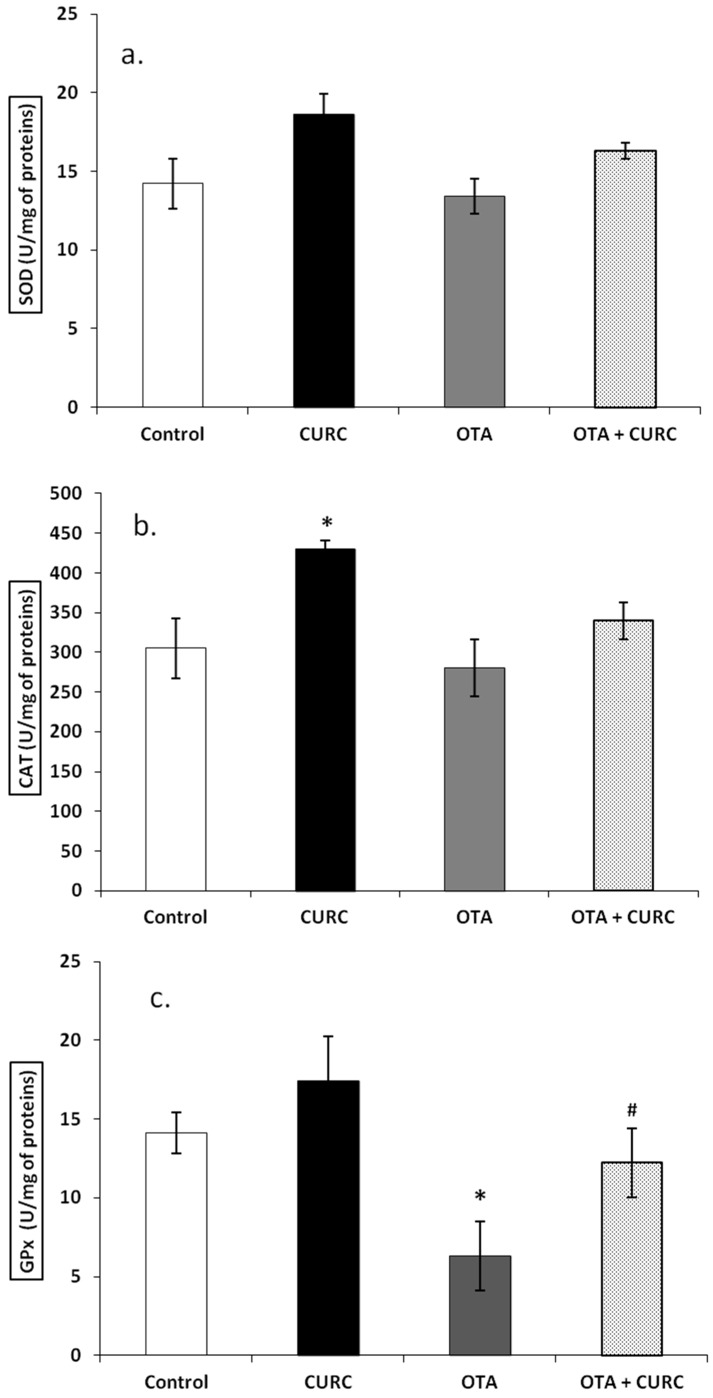
Effects of Curcumin (CURC) on superoxide dismutase (SOD), catalase (CAT) and glutathione peroxidase (GPx) activities expressed as unit for milligrams of proteins (U/mg of proteins) in renal tissue of experimental groups after 14 days of treatment. (**a**) Renal SOD activity, (**b**) Renal CAT activity, (**c**) Renal GPx activity. Control group (Control); Curcumin group (CURC); Ochratoxin A group (OTA); Curcumin plus Ochratoxin A group (CURC + OTA). Data are expressed as mean ± standard deviation (SD) of *n* = 6 rats. OTA treatment significantly decreased GPx enzyme activity, while co-administration with CURC significantly restored this effect (*p < 0.05 versus Control; #*p* < 0.05 versus OTA).

There was no significant difference in the SOD activity of the renal tissue in the different experimental groups after 14 days of treatment. Actually, the SOD value was 14.22 ± 1.62 in the Control group, 13.40 ± 1.12 in the OTA group, 18.64  ±  1.31 in the CURC group and 16.31 ± 0.51 in the OTA plus CURC group, respectively (Figure 2a). Also, the CAT activity of the renal tissue did not change in the OTA group or in the OTA plus CURC group in comparison to the Control group after 14 days of treatment. Actually, the CAT value was 280.20 ± 36.11 in the OTA group, 339.81 ± 23.21 in the OTA plus CURC group compared to 305.10 ± 38.12 in the Control group. A significant increase in CAT enzyme activity was found when CURC was used alone, in fact, the CAT value was 430.12  ±  10.51 in the CURC group compared to 305.10 ± 38.12 in the Control group (*p < 0.05) (Figure 2b). Instead, OTA significantly reduced the GPx activity. Actually, the GPx value was changed from 14.11 ± 1.31 in the Control group to 6.32 ± 2.22 in the OTA group (*p < 0.05) and, when the OTA group was treated with CURC, we observed a good recovery of the GPx value (12.23  ±  2.21 OTA plus CURC compared to 6.32 ± 2.22 in the OTA group, #*p* < 0.05). When CURC was used alone, no change in the GPx activity was observed (17.42 ± 2.82). (Figure 2c).

### 3.5. Lipid Peroxidation

**Figure 3:**
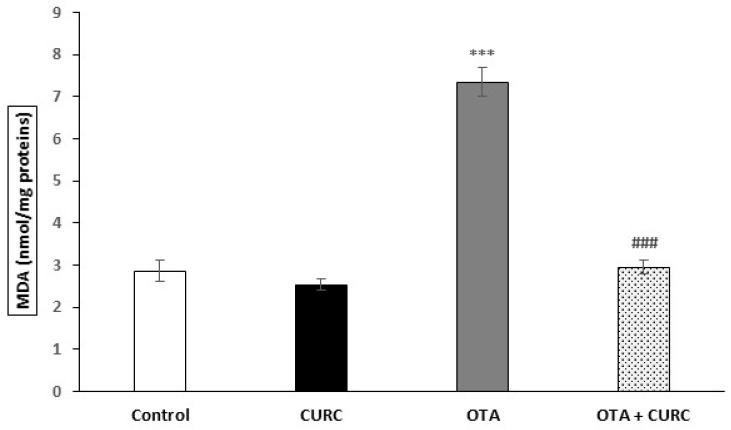
Effects of Curcumin (CURC) used alone or in association with Ochratoxin A (OTA) on lipid peroxidation measured by malondialdehyde (MDA), expressed in nmole of MDA for milligrams of proteins in rat kidney after 14 days of treatment. Control group (Control); Curcumin group (CURC); Ochratoxin A group (OTA); Curcumin plus Ochratoxin A group (CURC + OTA). Results are expressed as mean ± standard deviation (SD) of *n* = 6 rats. OTA treatment significantly increased MDA levels, while co-administration with CURC prevented this effect.  (***p < 0.001 versus Control; ^###^*p* < 0.001 versus OTA).

MDA levels in the renal tissues were significantly increased in the OTA compared to the Control group (2.86 ± 0.26 Control compared to 7.35 ± 0.33 OTA (***p < 0.001). Groups which received CURC with OTA showed a significant decrease in MDA levels compared with the OTA group. Indeed, the MDA values switched from 7.35 ± 0.33 (OTA) to 2.95 ± 0.17 (OTA + CURC) (###*p* < 0.001). When CURC was used alone, there was no change in MDA levels compared to the control group (2.53 ± 0.14 CURC compared to 2.86 ± 0.26 Control group) (Figure 3).

### 3.6. Histopathological Examination

**Figure 4:**
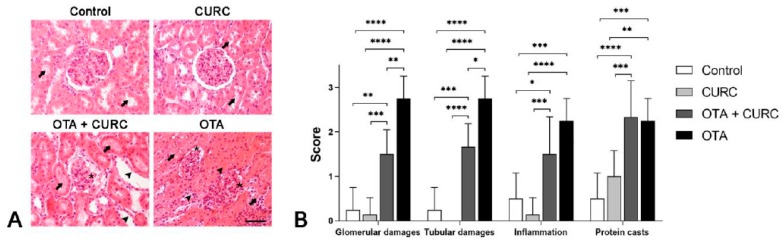
Renal histopathological evaluation of study groups. Control group (Control); Curcumin group (CURC); Ochratoxin A group (OTA); Curcumin plus Ochratoxin A group (CURC + OTA). (**A**) Histopathological findings obtained from light microscopy analysis of hematoxylin–eosin-stained kidney tissues (magnification, 40×; scale bars, 50 μm). Rats of the Control group and CURC groups showed a small amount of intratubular proteinaceous material (arrow). Rats treated with both OTA and CURC showed segmental necrosis of the glomerulus (asterisk) and abundant proteinaceous material in Bowman’s spaces and tubules lumen (arrows) associated with severe tubular epithelial cell atrophy and necrosis (arrow heads). OTA-treated rats showed severe global reduction of Bowman’s space (asterisks), abundant proteinaceous material in Bowman’s spaces and tubules lumen (arrows) associated with severe tubular epithelial cell atrophy and necrosis (arrow heads). (**B**) Severity scores of glomerular damage, tubular damage, inflammation and the presence of proteinaceous material evaluated on hematoxylin–eosin-stained sections for each group. Asterisks represent statistical differences between groups (*p < 0.05, **p < 0.01, ***p < 0.001, ****p < 0.0001).

Rat kidneys of the Control and CURC-treated groups appeared to be in a normal state with a mild accumulation of intratubular proteinaceous material. Only in one case of the Control group and in one case of the CURC treated group did we observe an interstitial scattered lymphocytic infiltration. No differences were noted between the Control and CURC treated groups. Instead, kidneys from the OTA-treated group showed a diffuse global reduction of Bowman’s space with segmental or global glomerular necrosis. Bowman’s space and tubules lumen contained abundant proteinaceous material, erythrocytes and, occasionally, sloughed necrotic epithelial cells. Tubular epithelial cells were often atrophic, degenerated, or necrotic. Interstice was multifocally expanded by a moderate lymphocytic infiltrate and by severe fibrosis (Figure 4a).

Rats treated with both OTA and CURC showed a significant reduction of glomerular (**p < 0.01) and tubular damage (**p < 0.01), compared with the OTA group. However, no significant differences were seen regarding inflammation and proteinaceous casts comparing the OTA + CURC with the OTA group. The group treated with OTA + CURC showed more severe glomerular damage (**p < 0.01), tubular damage (***p < 0.001), inflammation (*p < 0.05) and proteinaceous casts (****p < 0.0001) compared with the Control group (Figure 4b).

**Figure 5:**
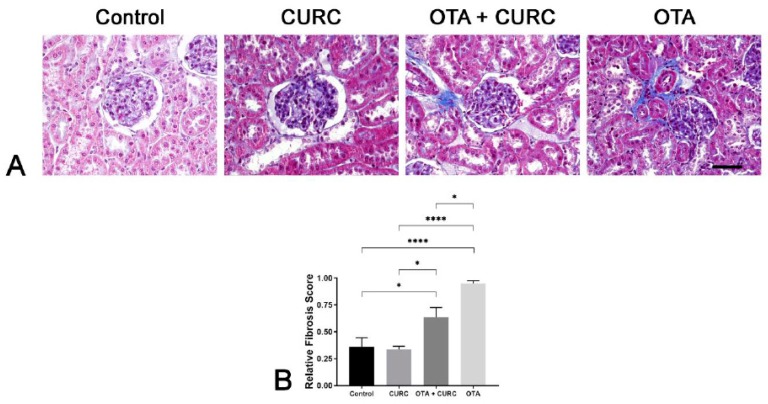
Renal histopathological evaluation of study groups. Control group (Control); Curcumin group (CURC); Ochratoxin A group (OTA); Curcumin plus Ochratoxin A group (CURC + OTA). (**A**) Histopathological findings obtained from light microscopy analysis of Masson’s trichrome-stained kidney tissues (magnification, 40×; scale bars, 50 μm). Rats of the Control group and CURC groups show normal fibers of connective tissue (blue) in the interstice. Rats of the OTA + CURC and OTA groups show an interstice severely expanded by abundant fibrous connective tissue (blue). (**B**) Severity of fibrosis evaluated on Masson’s trichrome-stained sections for each group. Asterisks denote statistical differences between groups (*p < 0.05, **p < 0.01, ***p < 0.001, ****p < 0.0001).

The group treated with OTA + CURC showed a less severe fibrosis (*p < 0.05) compared with the OTA group, but this change remains more severe than that observed in the Control group (*p < 0.05) (Figure 5a,b).

## 4. Discussion

OTA is a well-known and widely spread mycotoxin all over the world, which exerts dangerous effects on human and animal health [28]. Therefore, it is crucial to examine the toxic effects of OTA and to protect from its damage. The kidney is one of the most vulnerable target organs of OTA-induced toxicity [29]. Despite the numerous strategies developed to reduce OTA-induced nephrotoxicity, this mycotoxin mechanism of action remains complex and unknown. However, in recent years, several in vivo and in vitro studies have recognized that the induction of the oxidative stress induced by OTA exposure is considered one of the mechanisms responsible for its toxicity [30,31,32,33]. Thus, the administration of antioxidants that counteract oxidative stress may protect kidneys from damage. Nephrotoxicity studies have confirmed the authentic roles of bioactive compounds to reduce chronic renal failure [27,34,35,36]. The present study investigated CURC effects on OTA-induced toxicity using an in vivo animal model. CURC is a polyphenolic diketone natural product, commonly known as turmeric, with a multiplicity of pharmacologic properties. CURC is known to exert anti-inflammatory and antioxidant effects in various pathologies [37]. However, the protective effects of curcumin against OTA-mediated nephrotoxicity have not yet been studied using a mouse model. Therefore, the aim of our study was to examine the protective effects of CURC on OTA-induced renal toxicity through the evaluation of renal function and its main antioxidant enzymatic activities. To increase the stability and intestinal absorption of CURC, we used corn oil as a solvent. Due to good intestinal absorption, CURC increased the body weights of rats intoxicated by OTA after 14 days of treatment and, therefore, demonstrated a protective effect on the body weight of rats, considering that body weight is a main indicator for the assessment of toxic impacts of various drugs or chemicals on animals [38]. Actually, in our previous works carried out on rats treated with OTA we have always found a significant decrease in body weight compared to the control group subjects, as index of toxicity [7,27]. It is well-known that oxidative stress occurs due to an imbalance between oxidant and antioxidant systems, which could be due to elevated free radical generation and decreased antioxidant activity. Several studies suggest that both in vitro and in vivo OTA exposure results in the overproduction of free radicals [22,32,33], causing damage to cell constituents such as membrane lipids. Therefore, the main antioxidant enzymes, such as CAT, SOD and GPx, play a crucial role in protection against oxidative stress [39]. The results obtained in this study, in accordance with our previous works [7,21,27], confirmed that the renal toxic effects of OTA, both on a functional and histopathological level, is mediated by oxidative stress. Actually, in this study we found an increase of MDA in the kidneys of rats treated with OTA, which is an index of membrane lipid damage, and a decrease mainly in the activity of GPx, in line with the study conducted by Bertelli, et al. [40,41]. Furthermore, this study, according to the studies of Palabiyik et al. [42] and Domijan et al. [43], allows us to hypothesize that the toxic action of OTA is partly linked to its modulation on enzymatic activities and partly to a direct action on the production of ROS. CURC has been documented as a good antioxidant agent by many researchers [44,45,46] and the use of antioxidants has been shown to reduce oxidative stress either by eliminating free radicals or by rectifying the activity of the antioxidant defense system [47]. Here, CURC significantly reduced values of the lipid peroxidation with a consequent attenuation of the adverse effects of the chronic stress induced by OTA on tissue damage. It also appears to exert a protective antioxidant action on the kidney by enhancing the main enzymatic activities, such as SOD and CAT, and reversing the inhibitory effects of oxidative stress induced by OTA on GPx activity. These results indicate that, at least, the anti-stress activity of CURC may be in part due to its antioxidant effect. Here, we also analyzed the functional nephrotoxicity indices, such as BUN and CREA. The increase in BUN is often associated with renal insufficiency while creatinine is commonly used as a renal function measure and is the first step in controlling the glomerular filtration rate (GFR) [48]. BUN and CREA values were elevated in rats treated with OTA. The CURC significantly reduced BUN and CREA values in the serum of rats treated with OTA and this, in accordance with several data in literature, could be due to a reduction in renal damage by the CURC [49,50,51]. The increase in CREA observed in rats treated with OTA is related to the GFR reduction, which is associated with oxygen free radical formation. Actually, oxidative stress can promote the formation of a variety of vasoactive mediators that can affect renal functions directly by causing renal vasoconstriction or decreasing the glomerular capillary ultrafiltration coefficient and, thus, reduce GFR [52]. Instead, CURC in association with OTA completely prevented this renal hemodynamic alteration. The reported histological modifications are comparable to that described in OTA-induced damage in rats [27,53]. We hypothesized that the OTA-dependent damage of the secondary foot processes of the podocytes may be the cause of the presence of proteins in the Bowman’s spaces and tubules lumen and may be the cause of body weight loss [27,53]. The mechanisms underlying the protective role of the antioxidants toward OTA-related tubular epithelial cell damage is still debatable. We hypothesized that antioxidants, such as CURC, may counteract the production of reactive oxygen species (e.g., superoxide anion, hydroxyl radical and peroxide) and the OTA-induced lipid peroxidation mediated by cytochrome P450 activity in producing OTA–Fe3+/2+ complexes [54,55]. Decreased collagen degradation through matrix metalloproteinase inhibition and increased collagen cross-links are considered key mechanisms leading to OTA-induced kidney cortex fibrosis [56]. Our results showed a recovery of fibrosis in the OTA plus CURC group compared with the OTA group, suggesting a CURC protective role on the fibrosis development also. The effects of CURC on renal damage have been investigated in animal models, both in vivo and in vitro. Chronic supplementation of CURC has been shown to protect renal damage in various chemically-induced nephrotoxicity and renal injury models [57]. Our morphologic results support the hypothesis that CURC, as other antioxidants [27], reduces glomerular and tubular damage, kidney inflammation and tubulointerstitial fibrosis in rats, potentially preserving kidney function. Our results confirmed that oxidative stress is involved in the OTA nephrotoxicity mechanism. Exposure to OTA induces negative effects and profound changes in kidney functions. CURC showed an improvement in kidney function, biochemical and antioxidant enzyme activities and MDA levels. These observations also were supported by morphologic results such as a reduction in glomerular and tubular damage, in inflammation and in tubulointerstitial fibrosis. Therefore, although further data are needed on the practical application of the CURC, adding it to feed could offset OTA contamination and limit animal exposure to this toxic mycotoxin, thus protecting animal and human health.

## Figures and Tables

**Table 1:** Body weight (B.W.) expressed in grams (g) in the different groups of rats to 0, 7 and 14 days of treatment. Control group (Control); Curcumin group (CURC); Ochratoxin A group (OTA); Curcumin plus Ochratoxin A group (CURC + OTA). Data are expressed as mean ± standard deviation (SD) of *n* = 6 rats. (*p < 0.05 versus Control; #*p* < 0.05 versus OTA).

Groups	b.w. (g) 0 Days	b.w. (g) 7 Days	b.w. (g) 14 Days
Control	255.50 ± 4.6	288.92 ± 7.0	315.60 ± 5.8
CURC	257.11 ± 5.3	292.50 ± 6.2	314.50 ± 5.6 ^#^
OTA	252.21 ± 5.8	248.91 ± 4.6 *	258.32 ± 4.9 *
CURC + OTA	258.20 ± 4.7	257.72 ± 6.1	272.31 ± 5.2 ^#^

**Table 2:** Serum biochemical parameters: Blood urea nitrogen (BUN) and Creatinine (CREA) expressed in mg/dL concentration values in the different groups of rats after 14 days of treatment. Control group (Control); Curcumin group (CURC); Ochratoxin A group (OTA); Curcumin plus Ochratoxin A group (CURC + OTA). Data are expressed as mean ± standard deviation (SD) of *n* = 6 rats. (*p < 0.05, ***p < 0.001 versus Control; #*p* < 0.05, ### *p* < 0.001 versus OTA).

Groups	BUN (mg/dL)	CREA (mg/dL)
Control	42.08 ± 2.12	0.27 ± 0.03
CURC	38.13 ± 2.25 *^;^^###^	0.24 ± 0.05 ^#^
OTA	57.54 ± 0.05 ***	0.40 ± 0.08 *
CURC + OTA	48.12 ± 0.05 *^; ###^	0.35 ± 0.02 *

## Data Availability

The data sets used in the current study are available from the corresponding author on reasonable request.

## References

[1] Pitt J.I., Basilico J.C., Abarca M.L., Lopez C. (2000). Mycotoxins and toxigenic fungi. Med. Mycol..

[2] Pfohl-Leszkowicz A., Manderville R.A. (2007). Ochratoxin A: An overview on toxicity and carcinogenicity in animals and humans. Mol. Nutr. Food Res..

[3] Streit E., Schatzmayr G., Tassis P., Tzika E., Marin D., Taranu I., Tabuc C., Nicolau A., Aprodu I., Puel O. (2012). Current situation of mycotoxin contamination and co occurrence in animal feed focus on Europe. Toxins.

[4] Covarelli L., Beccari G., Marini A., Tosi L. (2012). A review on theoccurrence and control of ochratoxigenic fungal species andochratoxin a in dehydrated grapes, non-fortified dessert wines and dried vine fruit in the mediterranean area. Food Control..

[5] Marín S., Cano-Sancho G., Sanchis V., Ramos A.J. (2018). The role of mycotoxins in the human exposome: Application of mycotoxin biomarkers in exposome-health studies. Food Chem. Toxicol..

[6] Khoury E.A., Atoui A. (2010). Ochratoxin A: General overview and actual molecular status. Toxins.

[7] Damiano S., Navas L., Lombari P., Montagnaro S., Forte I.M., Giordano A., Florio S., Ciarcia R. (2018). Effects of δ-tocotrienol on ochratoxin A-induced nephrotoxicity in rats. J. Cell. Physiol..

[8] Bui-Klimke T., Wu F. (2014). Evaluating weight of evidence in the mystery of balkan endemic nephropathy. Risk Anal..

[9] Pavlovi’c N.M. (2013). Balkan endemic nephropathy—Current status and future perspectives. Clin. Kidney J..

[10] Dall’Asta C., Galaverna G., Bertuzzi T., Moseriti A., Pietri A., Dossena A., Marchelli R. (2010). Occurrence of ochratoxin A in raw ham muscle, salami and dry-cured hamfrom pigs fed with contaminated diet. Food Chem..

[11] Bertuzzi T., Gualla A., Morlacchini M., Pietri A. (2013). Direct and indirect contamination with ochratoxin A of ripened pork products. Food Control.

[12] Perši N., Pleadin J., Kovačević D., Scortichini G., Milone S. (2014). Ochratoxin A in raw materials and cooked meat products made from OTA-treated pigs. Meat Sci..

[13] Gallo A., Giuberti G., Frisvad J.C., Bertuzzi T., Nielsen K.F. (2015). Review on mycotoxin issues in ruminants: Occurrence in forages, effects of mycotoxin ingestion on health status and animal performance and practical strategies to counteract their negative effect. Toxins.

[14] Coronel M.B., Sanchis V., Ramos A.J., Marin S. (2010). Review. Ochratoxin A: Presence in human plasma and intake estimation. Food Sci. Technol. Int..

[15] Ak T., Gülçin I. (2008). Antioxidant and radical scavenging properties of curcumin. Chem. Biol. Interact..

[16] Edwards R.L., Luis P.B., Varuzza P.V., Joseph A.I., Presley S.H., Chaturvedi R., Schneider C. (2017). The anti-inflammatory activity of curcumin is mediated by its oxidative metabolites. J. Biol. Chem..

[17] De R., Kundu P., Swarnakar S., Ramamurthy T., Chowdhury A., Nair G.B., Mukhopadhyay A.K. (2009). Antimicrobial activity of curcumin against Helicobacterpylori isolates from India and during infections in mice. Antimicrob. Agents Chemother..

[18] Khazaei Koohpar Z., Entezari M., Movafagh A., Hashemi M. (2015). Anticancer activity of curcumin on human breast adenocarcinoma: Role of mcl-1gene. Iran J. Cancer Prev..

[19] Trujillo J., Chirino Y.I., Molina-Jijón E., Andérica-Romero A.C., Tapia E., Pedraza-Chaverrí J. (2013). Renoprotective effect of the antioxidant curcumin: Recent findings. Redox Biol..

[20] Avci H., Epikmen E.T., Ipek E., Tunca R., Birincioglu S.S., Akşit H., Sekkin S., Akkoç A.N., Boyacioglu M. (2017). Protective effects of silymarin and curcumin on cyclophosphamide-induced cardiotoxicity. Exp. Toxicol. Pathol..

[21] Damiano S., Puzio M.V., Squillacioti C., Mirabella N., Zona E., Mancini A., Borrelli A., Astarita C., Boffo S., Giordano A. (2018). Effect of rMnSOD on sodium reabsorption in renal proximal tubule in ochratoxin A-treated rats. J. Cell. Biochem..

[22] Ciarcia R., Damiano S., Squillacioti C., Mirabella N., Pagnini U., Florio A., Severino L., Capasso G., Borrelli A., Mancini A. (2016). Recombinant mitochondrial manganese containing superoxide dismutase protects against ochratoxin A-induced nephrotoxicity. J. Cell. Biochem..

[23] Sun Y., Oberley L.W., Li Y. (1988). A simple method for clinical assay of superoxide dismutase. Clin. Chem..

[24] Sinha A.K. (1972). Colorimetric assay of catalase. Anal. Biochem..

[25] Akerboom T.P., Sies H. (1981). Assay of glutathione, glutathione disulfide, and glutathione mixed disulfides in biological samples. Methods Enzimol..

[26] Ghanizadeh-Kazerouni E., Franklin C.E., Seebacher F. (2017). Living in flowing water increases resistance to ultraviolet B radiation. J. Exp. Biol..

[27] Damiano S., Iovane V., Squillacioti C., Mirabella N., Prisco F., Ariano A., Amenta M., Giordano A., Florio S., Ciarcia R. (2020). Red orange and lemon extract prevents the renal toxicity induced by ochratoxin A in rats. J. Cell. Physiol..

[28] Kőszegi T., Poór M. (2016). Ochratoxin A: Molecular interactions, mechanisms of toxicity and prevention at the molecular level. Toxins.

[29] Lee H.J., Pyo M.C., Shin H.S., Ryu D., Lee K.W. (2018). Renal toxicity through AhR, PXR, and Nrf2 signaling pathway activation of ochratoxin A-induced oxidative stress in kidney cells. Food Chem. Toxicol..

[30] El-Haleem M.R., Kattaia A.A., El-Baset S.A., Mostafa H.S. (2016). Alleviative effect of myricetin on ochratoxin A-induced oxidative stress in rat renal cortex: Histological and biochemical study. Histol. Histopathol..

[31] Abdel-Wahhab M.A., Aljawish A., El-Nekeety A.A., Abdel-Aziem S.H., Hassan N.S. (2017). Chitosan nanoparticles plus quercetin suppress the oxidative stress, modulate DNA fragmentation and gene expression in the kidney of rats fed ochratoxin A contaminated diet. Food Chem. Toxicol..

[32] Costa J.G., Saraiva N., Guerreiro P.S., Louro H., Silva M.J., Miranda J.P., Castro M., Batinic-Haberle I., Fernandes A.S., Oliveira N.G. (2016). Ochratoxin A-induced cytotoxicity, genotoxicity and reactive oxygen species in kidney cells: An integrative approach of complementary endpoints. Food Chem. Toxicol.

[33] Periasamy R., Kalal I.G., Krishnaswamy R., Viswanadha V. (2016). Quercetin protects human peripheral blood mononuclear cells from OTA-induced oxidative stress, genotoxicity, and inflammation. Environ. Toxicol..

[34] Hassan S.S., Rizk A., Thomann C., Motawie A., Abdelfattah S., Ahmad Z. (2019). Preconditioning with atorvastatin against renal ischemia-reperfusion injury in nondiabetic versus diabetic rat. Can. J. Physiol. Pharmacol..

[35] Changizi-Ashtiyani S., Seddigh A., Najafi H., Hossaini N., Avan A., Akbary A., Manian M., Nedaeinia R. (2017). *Pimpinella anisum L*. ethanolic extract ameliorates the gentamicin- induced nephrotoxicity in rats. Nephrology (Carlton).

[36] Damiano S., Lombari P., Salvi E., Papale M., Giordano A., Amenta M., Ballistreri G., Fabroni S., Rapisarda P., Capasso G. (2019). A red orange and lemon by-products extract rich in anthocyanins inhibits the progression of diabetic nephropathy. J. Cell. Physiol..

[37] García-Niño W.R., Pedraza-Chaverrí J. (2014). Protective effect of curcumin against heavy metals-induced liver damage. Food Chem. Toxicol..

[38] Wang C., Lu J., Zhou L., Li J., Xu J., Li W., Zhang L., Zhong X., Wang T. (2016). Effects of long-term exposure to zinc oxide nanoparticles on development, zinc metabolism and biodistribution of minerals (Zn, Fe, cu, Mn) in mice. PLoS ONE.

[39] Blake D.R., Allen R.E., Lunec J. (1987). Free radicals in biological systems? a review orientated to inflammatory processes. Br. Med. Bull..

[40] Bertelli A.A., Migliori M., Filippi C., Gagliano N., Donetti E., Panichi V., Scalori V., Colombo R., Mannari C., Tillement J.P. (2005). Effect of ethanol and red wine on ochratoxin a-induced experimental acute nephrotoxicity. J. Agric. Food Chem..

[41] Dai J., Park G., Wright M.W., Adams M., Akman S.A., Manderville R.A. (2002). Detection and characterization of a glutathione conjugate of ochratoxin A. (2002). Chem. Res. Toxicol..

[42] Palabiyik S.S., Erkekoglu P., Zeybek N.D., Kizilgun M., Baydar D.E., Sahin G., Giray B.K. (2013). Protective effect of lycopene against ochratoxin A induced renal oxidative stress and apoptosis in rats. Exp. Toxicol. Pathol..

[43] Domijan A.M., Peraica M., Vrdoljak A.L., Radic B., Zlender V., Fuchs R. (2007). The involvement of oxidative stress in ochratoxin A and fumonisin B1 toxicity in rats. Mol. Nutr. Food Res..

[44] Farkhondeh T., Samarghandian S., Samini F. (2016). Antidotal effects of curcumin against neurotoxic agents: An updated review. Asian Pac. J. Trop. Med..

[45] Iqbal M., Sharma S.D., Okazaki Y., Fujisawa M., Okada S. (2003). Dietary supplementation of curcumin enhances antioxidant and phase II metabolizing enzymes in ddY male mice: Possible role in protection against chemical carcinogenesis and toxicity. Pharmacol. Toxicol..

[46] Nazari Q.A., Takada-Takatori Y., Hashimoto T., Imaizumi A., Izumi Y., Akaike A., Kume T. (2014). Potential protective effect of highly bioavailable curcumin on an oxidative stress model induced by microinjection of sodium nitroprusside in mice brain. Food Funct..

[47] Samini F., Samarghandian S., Borji A., Mohammadi G., Bakaian M. (2013). Curcumin pretreatment attenuates brain lesion size and improves neurological function following traumatic brain injury in the rat. Pharmacol. Biochem. Behav..

[48] Mitchell H.R., Kline W. (2006). Core curriculum in nephrology, renal function testing. Am. J. Kidney Dis..

[49] Najafi H., Ashtiyani S.C., Sayedzadeh S.A., Mohamadi Yarijani Z., Fakhri S. (2015). Therapeutic effects of curcumin on the functional disturbances and oxidative stress induced by renal ischemia/reperfusion in rats. Avicenna J. Phytomed..

[50] Guzel S., Sahinogullari Z.U., Canacankatan N., Antmen S.E., Kibar D., Coskun Yilmaz B. (2019). Potential renoprotective effects of silymarin against vancomycin-induced nephrotoxicity in rats. Drug Chem. Toxicol..

[51] Hassan S.M.S., Youakim M.F., Rizk A.A.E., Thomann C., Ahmad Z. (2017). Does silybin protect against toxicity induced by polymyxin E in rat kidney?. Neurourol. Urodyn..

[52] Craven P.A., Melhem M.F., DeRubertis F.R. (1992). Thromboxane in the pathogenesis of glomerular injury in diabetes. Kidney Int..

[53] Abdu S., Ali A., Ansari S. (2011). Cytotoxic effect of ochratoxin a on the renal corpuscles of rat kidney: Could ochratoxin a cause kidney failure?. Histol. Histopathol..

[54] Sorrenti V., Di Giacomo C., Acquaviva R., Barbagallo I., Bognanno M., Galvano F. (2013). Toxicity of ochratoxin A and its modulation by antioxidants: A review. Toxins (Basel).

[55] Narayan M.S., Naidu K.A., Ravishankar G.A., Srinivas L., Venkataraman L.V. (1999). Antioxidant effect of anthocyanin on enzymatic and non-enzymatic lipid peroxidation. Prostaglandins Leukot. Essent. Fat. Acids.

[56] Gagliano N., Torri C., Donetti E., Grizzi F., Costa F., Bertelli A.A., Migliori M., Filippi C., Bedoni M., Panichi V. (2005). Ochratoxin A-induced renal cortex fibrosis and epithelial-to-mesenchymal transition: Molecular mechanisms of ochratoxin A-injury and potential effects of red wine. Mol. Med..

[57] Ghelani H., Razmovski-naumovski V., Chang D., Nammi S. (2019). Chronic treatment of curcumin improves hepatic lipid metabolism and alleviates the renal damage in adenine-induced chronic kidney disease in Sprague-Dawley rats. BMC Nephrol..

